# Supplementary material for: Lycium barbarum Glycopeptide Inhibits Colorectal Cancer Cell Proliferation via Activating p53/p21 Pathway and Inducing Cellular Senescence
Source: Int J Mol Sci. 2025 Jul 23;26(15):7091. doi: 10.3390/ijms26157091 (PMC12345642; doi:10.3390/ijms26157091)
Supplement: Supplementary file 1 [file ijms-26-07091-s001.zip › ijms-3738521-supplementary.pdf]

***Lycium Barbarum* Glycopeptide Inhibits Colorectal Cancer Cell Proliferation via Activating p53/p21 Pathway and Inducing Cellular Senescence**

This supplemental file includes:

Figure S1 Effect of LbGP on the proliferation of non-cancerous cell lines.

Figure S2 p53 inhibitor Pifithrin-α prevents the anti-tumor effect of LbGP.

Figure S3 LbGP does not affect major organ weights in treated mice.

Figure S4 LbGP increases γ-H2AX expression in tumor tissues.

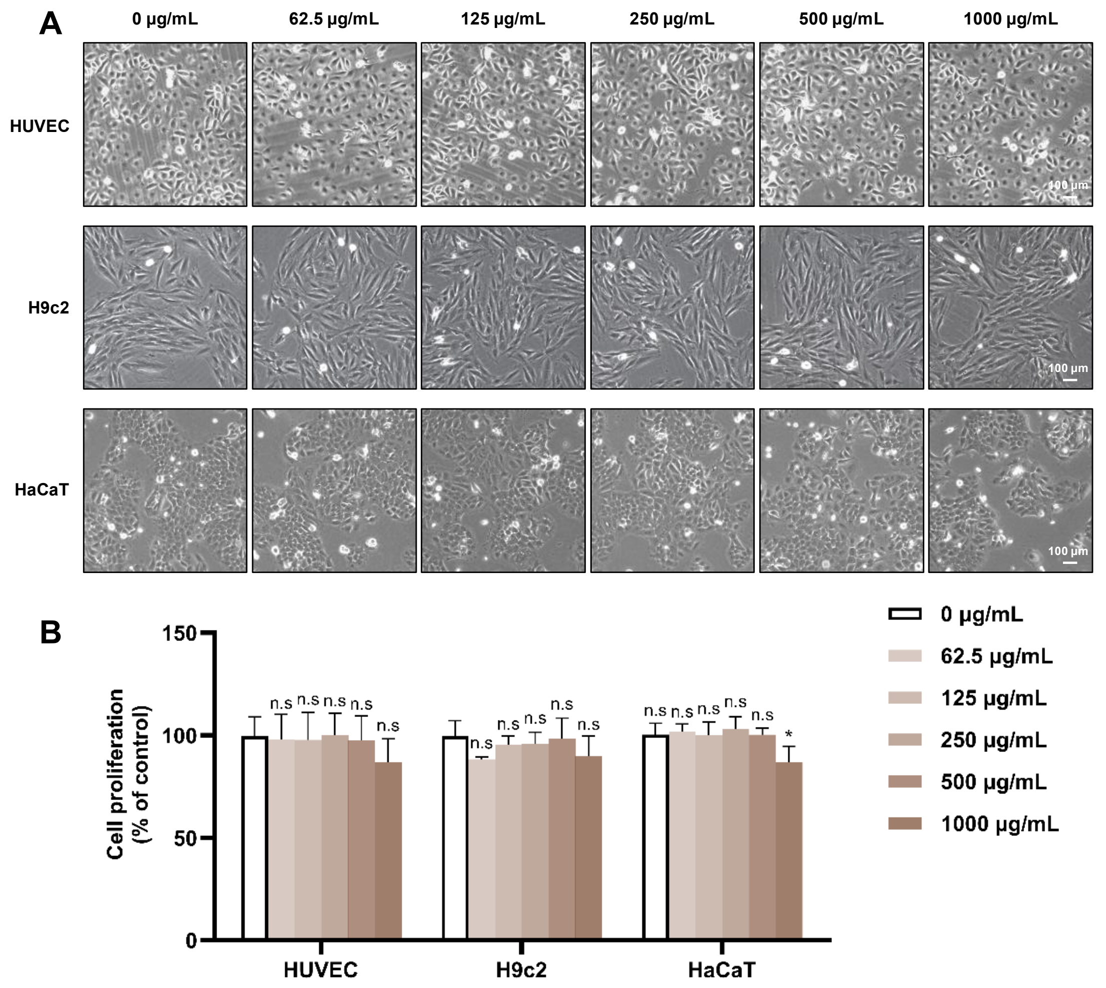

Figure S1 Effect of LbGP on the proliferation of non-cancerous cell lines.

A. Morphology and viable cell count of HUVEC, H9c2, and HaCaT cells after 72 h treatment with various concentrations of LbGP (200×; scale bar, 100 μm; Leica inverted microscope).

B. Relative proliferation rates of HUVEC, H9c2, and HaCaT cells after 72 h treatment with LbGP.

n = 3. *n.s.* = not significant, *$p < 0.05$, compared to control (0 μg/mL).

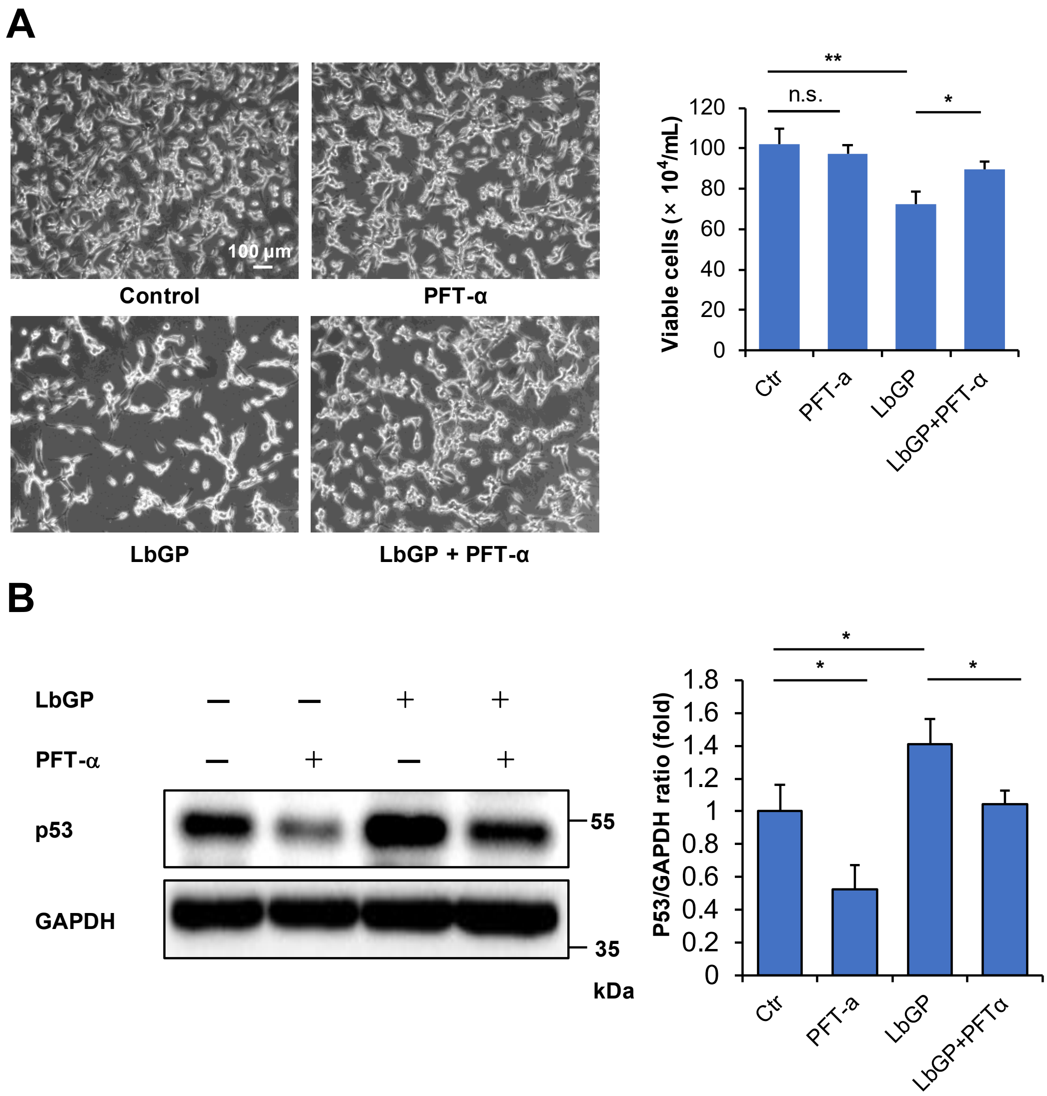

Figure S2 p53 inhibitor Pifithrin-α prevents the anti-tumor effect of LbGP.

A. Morphology and viable cell count of CT26 cells after 48 h treatment with LbGP (250 µg/mL) and the p53 inhibitor Pifithrin-α (PFTα, 10 µM) , (200×; scale bar, 100 µm; Leica inverted microscope).The p53 inhibitor Pifithrin-α (PFTα, 10 µM).

B. Western blot and quantification of p53 expression after treatment with LbGP (250 µg/mL and the p53 inhibitor Pifithrin-α (PFTα, 10 µM).

n = 3. *n.s.*, not significant, *p < 0.05; **p < 0.01; compared to the control.

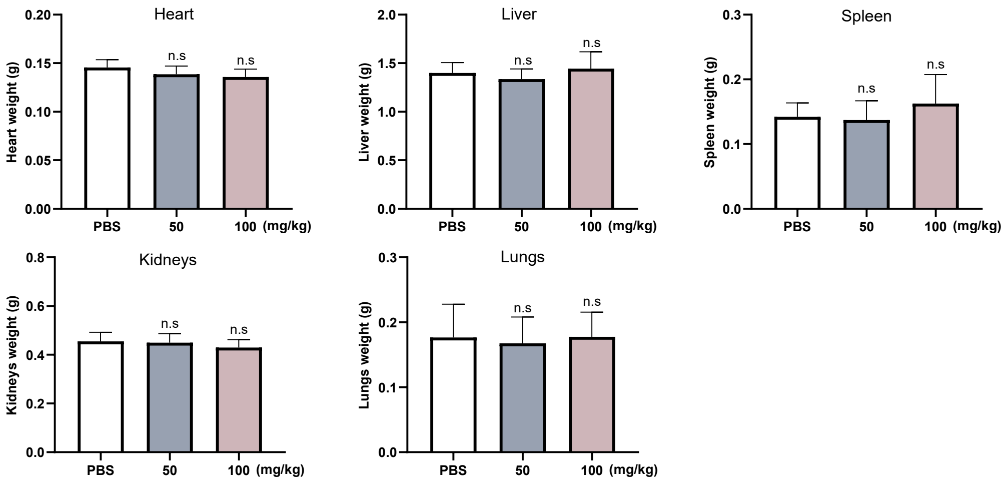

Figure S3 LbGP does not affect major organ weights in treated mice.

Organ weights of the heart, liver, spleen, lungs, and kidneys were measured on day 21. No significant differences were observed between the LbGP-treated group (100 mg/kg) and the PBS control group.

n = 8 per group; data represent mean ± SD. *n.s.*, not significant, compared to PBS.

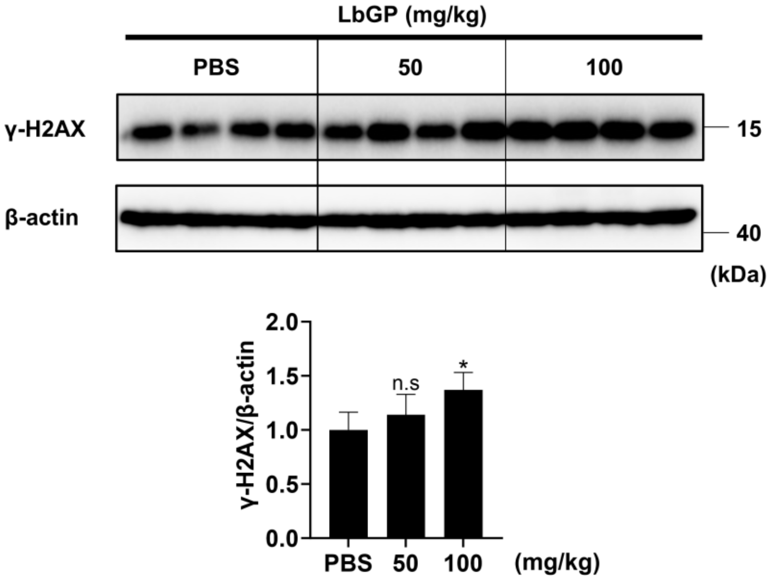

Figure S4 LbGP increases γ-H2AX expression in tumor tissues.

Western blot showing increased γ-H2AX levels in tumor tissues on day 21 following 100 mg/kg LbGP treatment. β-actin served as the loading control.

n = 8 per group; mean ± SD. *n.s.*, not significant; $*p < 0.05$, compared to PBS.
